# HCV-coinfection is related to an increased HIV-1 reservoir size in cART-treated HIV patients: a cross-sectional study

**DOI:** 10.1038/s41598-019-41788-9

**Published:** 2019-04-03

**Authors:** Maria Rosa López-Huertas, Claudia Palladino, Marta Garrido-Arquero, Beatriz Esteban-Cartelle, Marta Sánchez-Carrillo, Paula Martínez-Román, Luz Martín-Carbonero, Pablo Ryan, Lourdes Domínguez-Domínguez, Ignacio De Los Santos, Sara De La Fuente Moral, José Miguel Benito, Norma Rallón, José Alcamí, Salvador Resino, Amanda Fernández-Rodríguez, Mayte Coiras, Verónica Briz, on behalf of the Multidisciplinary Group of viral coinfection HIV/Hepatitis (COVIHEP), Alfonso Ángel-Moreno, Laura Bermejo-Plaza, Otilia Bisbal, Oscar Brochado-Kith, Juan Miguel Castro-Álvarez, Guillermo Cuevas, Victorino Diez-Viñas, Marta Gálvez-Charro, Lucio García-Fraile, Alicia Gómez-Sanz, María Lagarde, Mariano Matarranz, Irene Mate-Cano, Mario Mayoral-Muñoz, María Muñoz-Muñoz, Federico Pulido, Rafael Rubio, Mireia Santacreu, Jesús Sanz-Sanz, Nuno Taveira, Jesús Troya, Isabel Cortegano, María Luisa Gaspar

**Affiliations:** 1 Department of Infectious Diseases, Instituto Ramón y Cajal de Investigación Sanitaria (IRYCIS), Madrid, Spain; 2 Research Institute for Medicines (iMed.ULisboa), Faculty of Pharmacy, Universidade de Lisboa, Lisbon, Portugal; 3 Unit of Viral Infection and Immunity, National Center for Microbiology, Institute of Health Carlos III, Majadahonda, Madrid, Spain; 4 Instituto de Investigación Sanitaria Hospital de la Paz (IdiPAZ), Madrid, Spain; 5 Department of Infectious Diseases, Infanta Leonor Hospital, Madrid, Spain; 6 Unidad VIH. Servicio de Medicina Interna. Instituto de Investigación Biomédica del Hospital Doce de Octubre (imas12), Madrid, Spain; 7 Servicio de Medicina Interna-Infecciosas. Hospital Universitario de La Princesa, Madrid, Spain; 8 Servicio de Medicina Interna. Hospital Puerta de Hierro, Madrid, Spain; 9 Instituto de Investigación Sanitaria Fundación Jiménez Díaz, Universidad Autónoma de Madrid (IIS-FJD, UAM), Madrid, Spain; 10 Hospital Universitario Rey Juan Carlos, Móstoles, Spain; 11 AIDS Immunopathology, Centro Nacional de Microbiología, Instituto de Salud Carlos III, Majadahonda, Madrid, Spain; 12 Inmunology Department, Centro Nacional de Microbiología, Instituto de Salud Carlos III, Madrid, Spain

## Abstract

In HIV-1/HCV-coinfected patients, chronic HCV infection leads to an increased T-lymphocyte immune activation compared to HIV-monoinfected patients, thereby likely contributing to increase HIV-1 reservoir that is the major barrier for its eradication. Our objective was to evaluate the influence of HCV coinfection in HIV-1 viral reservoir size in resting (r) CD4+ T-cells (CD25-CD69-HLADR-). Multicenter cross-sectional study of 97 cART-treated HIV-1 patients, including 36 patients with HIV and HCV-chronic co-infection without anti-HCV treatment, 32 HIV patients with HCV spontaneous clearance and 29 HIV-monoinfected patients. rCD4+ T-cells were isolated and total DNA was extracted. HIV viral reservoir was measured by Alu-LTR qPCR. Differences between groups were calculated with a generalized linear model. Overall, 63.9% were men, median age of 41 years and Caucasian. Median CD4+ and CD8+ T-lymphocytes were 725 and 858 cells/mm^3^, respectively. CD4+ T nadir cells was 305 cells/mm^3^. Proviral HIV-1 DNA size was significantly increased in chronic HIV/HCV-coinfected compared to HIV-monoinfected patients (206.21 ± 47.38 *vs*. 87.34 ± 22.46, respectively; *P* = 0.009), as well as in spontaneously clarified HCV co-infected patients when compared to HIV-monoinfected individuals (136.20 ± 33.20; *P* = 0.009). HIV-1/HCV co-infected patients showed a larger HIV-1 reservoir size in comparison to HIV-monoinfected individuals. This increase could lead to a greater complexity in the elimination of HIV-1 reservoir in HIV-1/HCV-coinfected individuals, which should be considered in the current strategies for the elimination of HIV-1 reservoir.

## Introduction

The human immunodeficiency virus (HIV-1) affects 36.9 million people worldwide accordingly to WHO-AIDS^1^. The combined antiretroviral therapy (cART) can successfully suppress HIV-1 viremia and significantly delays the progression of the disease^2^. However, the infection remains currently incurable and lifetime treatment is required due to the viral persistence in latent reservoirs that are not accessible to cART and not detectable by the immune system^3,4^. During HIV-1 replication, the viral genome is stably integrated in the host cell genome but viral genes are not expressed at significant levels in resting cells due to the absence of cellular transcription factors. This long-life latent reservoir is established *in vivo* very early after infection^5^ and consists mainly of several populations of memory resting (r) CD4 T-cells including stem central memory (T_SCM_), central memory (T_CM_), effector memory (T_EM_) and transitional memory (T_TM_) cells^6,7^. Once the infected cells become activated, the viral production starts^4,7^. The reservoir size is very small in patients on cART, estimated as 10–100 replication-competent latent provirus per million rCD4 T-cells^8^. However, it is sufficient to refuel viral replication after cART interruption^9^. Viral reservoir is also very stable and does not fade with time even with constant cART^10^.

Hepatitis C virus (HCV) infection is also a major health problem with 71 million people chronically infected^11^. Although HCV is primarily a hepatotropic virus, chronic HCV infection presents other important extrahepatic sites for propagation, including peripheral blood mononuclear cells (PBMCs). As CD4+ T cells are the primary site for HIV-1 replication, the co-infection of the same cell could lead to a complex interaction between both viruses^12–15^.

Due to shared routes of transmission, HCV co-infection in people living with HIV-1 affects up to 6.2% of HIV-infected individuals^16^. Spontaneous HCV clearance occurs in 10–15% of acute infections in HCV-monoinfected individuals but only in less than 10% of HIV-1 infected patients^17^. A better understanding of the impact of HCV infection on HIV-infected patients is essential to adequately manage HIV/HCV coinfected patients. In fact, important differences arise from HIV-monoinfected individuals from those co-infected with other pathogens, such as the high levels of immune activation and premature immune senescence that occur in patients co-infected with cytomegalovirus (CMV)^18–21^, herpesviruses^22^, *Mycobacterium tuberculosis* (TB)^23^ or with HCV^24^. In HIV/HCV coinfection, this may be due to the enhanced IL-10 production that occurs after HCV infection^25^. Interestingly, coinfection of HIV-1 with other viruses that induce immune activation, such as CMV, increases the size of the viral reservoir during the first steps of HIV-1 infection^26^. But this remains uncertain in the case of patients co-infected with HCV.

Consequently, in this work we analyzed the effect of HCV infection in the size of HIV-1 reservoir using rCD4+ T cells isolated from treatment-naive HCV co-infected individuals on cART.

## Results

### Study Patients

Patients were categorized according to their HCV status. In HIV+ monoinfected group, patients had never been in contact with HCV. The HIV+/HCV− group was formed with HIV+ patients who had been exposed to HCV but experienced HCV spontaneous viral clearance during the first 6 months after HCV infection. Finally, the HIV+/HCV+ group was constituted by patients with HIV on cART and active HCV-chronic infection naïve to any HCV treatment.

Epidemiological and clinical characteristics are summarized in Table 1. Patients were Caucasian, with a median age of 50 years [Interquartile range (IQR): 45–54] and 63.9% (n = 62) were men. The median HIV-1 infection time was 19.9 years (IQR: 8.8–26.6); significantly shorter in HIV+ individuals (15.1 years) than in HIV+/HVC− (21.7 years) or chronic HIV+/HVC+ (22.8 years) (*P* = 0.035). Patients with CDC category C status were almost one-fourth of the study group (23.7%), being less represented in HIV+ individuals (13.8%) than in HIV+/HVC− (28.1%) or chronic HIV+/HVC+ (27.8%), but this difference was not statistically significant. All patients presented a Fiebig stage ≥ V when initiated cART. Most of the patients who spontaneously cleared HCV or those with chronic HIV+/HCV+ acquired HIV-1 through intravenous drug use [62.5% (n = 20) and 58.3% (n = 21), respectively]. By contrast, none of HIV-monoinfected patients acquired HIV infection through parenteral route (*P* = 0.017). Thirty-seven percent of the patients were receiving a cART regimen based on integrase inhibitors and 31% on NNRTIs. More than 20% of patients were receiving dual or monotherapy based on protease inhibitors. Coinfection with CMV was also studied in all patients groups, observing a prevalence of 27.6%,46.9% and 44.4% in HIV-monoinfected, HIV+/HCV− and HIV+/HCV+ patients, respectively.

**Table 1:** Epidemiological Characteristics of the Study Population.

	Total (n = 97)	HIV+ (n = 29)	HIV+/HCV−(n = 32)	HIV+/HCV+ (n = 36)	*P*
**Median Age**, years [median (IQR)]	50 (45–54)	48 (38–54)	52 (48–55)	52 (44–54)	0.372
**Weight**, kg [median (IQR)]	70 (61–80)	75 (67–84)	76 (64–83)	62 (55–73)	0.002*
**Height**, cm [median (IQR)]	168 (162–174)	168 (163–173)	169 (164–175)	167 (160–174)	0.512
**BMI**, kg/m^2^ [median (IQR)]	24 (22–27)	26 (23–28)	25 (22–28)	23 (21–25)	0.012**
**Male**, n (%)	62 (63.9)	18 (62.1)	21 (65.6)	23 (63.9)	0.959
**Time of HIV infection**, years [median (IQR)]	19.9 (8.8–26.6)	15.1 (6.1–22.0)	21.7 (12.2–26.6)	22.8 (9.5–8.7)	0.035 ***
**Transmission route**, n (%)					
IDUs	41 (42.3)	0	20 (62.5)	21 (58.3)	*0.017*
MSM	27 (27.8)	13 (44.8)	6 (18.8)	8 (22.2)	
MSW	17 (17.5)	12 (41.4)	3 (9.4)	2 (5.5)	
Others	12 (12.3)	3 (9.4)	3 (9.4)	5 (14.1)	
**CDC category**, n (%)					
A	51 (52.5)	18 (62.1)	16 (50.0)	17 (47.2)	0.219
B	19 (19.6)	4 (13.8)	6 (18.8)	9 (25.0)	
C	23 (23.7)	4 (13.8)	9 (28.1)	10 (27.8)	
Unknown	4 (4.1)	3 (10.3)	1 (3.1)	0	
**cART regimen**, n (%)					
NNRTIs	30 (30.9)	8 (27.6)	13 (40.6)	9 (25.0)	0.232
PIs	10 (10.3)	2 (6.9)	4 (12.5)	4 (11.1)	
INIs	36 (37.1)	11 (37.9)	11 (34.4)	14 (38.9)	
Dual therapy	16 (16.5)	6 (20.7)	4 (12.5)	6 (16.7)	
Monotherapy	5 (5.2)	2 (6.9)	0	3 (8.3)	
**CMV Infection**, n, (%)					
Yes	39 (40.2)	8 (27.6)	15 (46.9)	16 (44.4)	0.309
No	29 (29.9)	12 (41.4)	6 (18.8)	11 (30.6)	
Unknown	29 (29.9)	9 (31.0)	11 (34.4)	9 (25.0)	
**IFNL3 (IL28B) genotype**, n (%)					
CC	53 (54.6)	16 (55.2)	24 (75.0%)	13 (37.1)	*0.008*
Non-CC	43 (44.4)	13 (44.8)	8 (25.0)	22 (61.9)	

Notes: BMI, body mass index; HIV, human immunodeficiency virus; HCV, hepatitis C virus; IDUs, Intravenous Drug Users; MSM, men who have sex with men; MSW, men who have sex with women; CDC, Centers for Disease Control and Prevention classification system for HIV infection; cART, combined antiretroviral therapy; NRTIs, nucleoside analogue reverse transcriptase inhibitors; NNRTIs, non-nucleoside reverse transcriptase inhibitors; PIs, protease inhibitors; INIs, integrase inhibitors; CMV, cytomegalovirus; %, percentage; IQR, interquartile range. CC: CC genotype. *Comparison of weight between groups performed by Mann-Whitney test: HIV+ group vs. HIV+/HCV+ group, P = 0.001; HIV+/HCV− group vs. HIV+/HCV+ group, P = 0.005. **Comparison of BMI between groups performed by Mann-Whitney test: HIV+ group vs. HIV+/HCV+ group, P = 004; HIV+/HCV− group vs. HIV+/HCV+ group, P = 0.050.

***Comparison of time of HIV infection between groups performed by Mann-Whitney test: HIV+ group vs. HIV+/HCV− group, P = 0.021; HIV+ group vs. HIV+/HCV+ group, P = 0.026.

Patients: ▪ HIV+ group: HIV-monoinfected patients that had never been in contact with HCV (negative PCR and HCV antibodies).

▪ HIV+/HCV− group: HIV patients who had been exposed to HCV but experienced spontaneous viral clearance during the first 6 months after HCV infection (negative PCR and positive HCV antibodies).

▪ HIV+/HCV+ group: patients with HIV and active HCV-chronic infection naïve to any HCV treatment (positive PCR and HCV antibodies).

Table 2 show the description on cell subpopulations. Median CD4 counts were similar among groups [952 cells/mm^3^ (747–1267) for HIV+ , 813 cells/mm^3^ (701–1005) for HIV+/HCV− and 757 cells/mm^3^ (524–1063) for HIV+/HVC+ subjects, *P* = 0.531]. No differences were either observed in CD8+ T lymphocytes [845 cells/mm^3^ (680–893) for HIV+ , 957 cells/mm^3^ (714–1219) HIV+/HCV− and 814 (638–1211) HIV+/HVC+ subjects, respectively, *P* = 0.541]. In addition, a tendency to a higher CD4:CD8 ratio was observed in HIV+ subjects [1.2 (1.1–1.4)] compared to patients exposed to HCV infection [0.8 (0.7–1.1) and 0.9 (0.7–1.1), for HIV+ HCV− and HIV+ HIV+ groups, respectively; (*P* = 0.096). Median CD4+ T nadir cells was 305 (IQR: 268–462) cells/mm^3^, and patients with spontaneous clearance showed the lowest nadir of CD4+ T-cells (*P* = 0.018) (Table 2).

**Table 2:** Clinical Characteristics of the Study Population.

	Total (n = 97)	HIV+ (n = 29)	HIV+/HCV−(n = 32)	HIV+/HCV+ (n = 36)	*P*
**CD4+ T cell count**, cells/µL [median (IQR)]	787 (625–1020)	952 (747–1267)	813 (701–1005)	757 (524–1063)	0.531
**CD4%** [median (IQR)]	37.4 (31.2–43.1)	42.5 (38.4–48.7)	33.5 (30.1–40.5)	34.7 (30.5–43.0)	0.064
**CD8 T cell count**, cells/µL [median (IQR)]	858 (732–1199)	845 (680–893)	957 (714–1219)	814 (638–1211)	0.541
**Nadir CD4**, cells/µL [median (IQR)]	N = 81 239 (134–327)	256 (202–374)	225 (110–266)	234 (81–320)	0.230
**Nadir CD4**% [median (IQR)]	N = 55 19.0 (12.0–28.1)	25.5 (18.0–28.8)	14.0 (7.0–19.5)	22.0 (14.0–32.0)	0.003*
**CD8%** [median (IQR)]	38.7 (36.7–43.0)	35.0 (26.5–38.2)	39.0 (32.1–43.1)	40.6 (36.5–43.2)	0.306
**CD4:CD8 Ratio**	0.9 (0.7–1.1)	1.2 (1.1–1.4)	0.8 (0.7–1.1)	0.9 (0.7–1.1)	0.096

Notes: HIV, human immunodeficiency virus; HCV, hepatitis C virus; %, percentage; IQR, interquartile range.

*Comparison of nadir CD4 (%) between groups performed by Mann-Whitney test: HIV+ group vs. HIV+/HCV− group, P = 0.001; HIV+/HCV− group vs. HIV+/HCV+ group, P = 0.010.

Patients: ▪ HIV+ group: HIV-monoinfected patients that had never been in contact with HCV (negative PCR and HCV antibodies).

▪ HIV+/HCV− group: HIV patients who had been exposed to HCV but experienced spontaneous viral clearance during the first 6 months after HCV infection (negative PCR and positive HCV antibodies).

▪ HIV+/HCV+ group: patients with HIV and active HCV-chronic infection naïve to any HCV treatment (positive PCR and HCV antibodies).

Table 3 show clinical characteristics related to HCV infection. In HIV+/HCV+ group, the most prevalent HCV genotypes were 1 (52.7%, n = 19) and 4 (30.3%, n = 11). Differences in fibrosis stages were significant between HIV/HCV groups (*P* = 0.033). Liver fibrosis, was determined by Fibroscan, in all patients ever in contact with HCV, such as the spontaneous clarifiers (SC) (n = 19) and HIV+/HCV+ (n = 26) co-infected patients. IFNL3 (IL28B) (rs12979860) CC genotype was found in 75% (n = 24) of HIV+/HCV− patients, 55.2% (n = 16) in HIV-monoinfected individuals and in 37.1% (n = 13) of co-infected patients (*P* = 0.012).

**Table 3:** Clinical characteristics related to HCV-infected patients.

	Total (n = 68)	HIV+/HCV−(n = 32)	HIV+/HCV+ (n = 36)	*P*
**HCV genotype**, n (%)				
GT1	19 (52.7)	—	19 (52.7)	—
GT2	1 (0.03)	—	1 (0.03)	—
GT3	2 (0.05)	—	2 (0.05)	—
GT4	11 (30.3)	—	11 (30.3)	—
Unknown	3 (0.08)	—	3 (0.08)	—
**Fibrosis**, n (%)				
F0	4 (11.9)	2 (6.3)	2 (5.6)	*0.033*
F1 (<6kPA)	41 (119.8)	17 (53.1)	24 (66.7)	
F2 (6–9kPa)	4 (11.1)	0	4 (11.1)	
F3 (>9–12kPa)	3 (8.3)	0	3 (8.3)	
F4 (>12kPa)	2 (5.6)	0	2 (5.6)	
Unknown	14 (43.4)	13 (40.6)	1 (2.8)	

Notes: HIV, human immunodeficiency virus; HCV, hepatitis C virus; %, percentage; kPa: kilopascals.

Patients: ▪ HIV+/HCV− group: HIV patients who had been exposed to HCV but experienced spontaneous viral clearance during the first 6 months after HCV infection (negative PCR and positive HCV antibodies).

▪ HIV+/HCV+ group: patients with HIV and active HCV-chronic infection naïve to any HCV treatment (positive PCR and HCV antibodies).

### HIV-1 proviral integration

**Figure 1:**
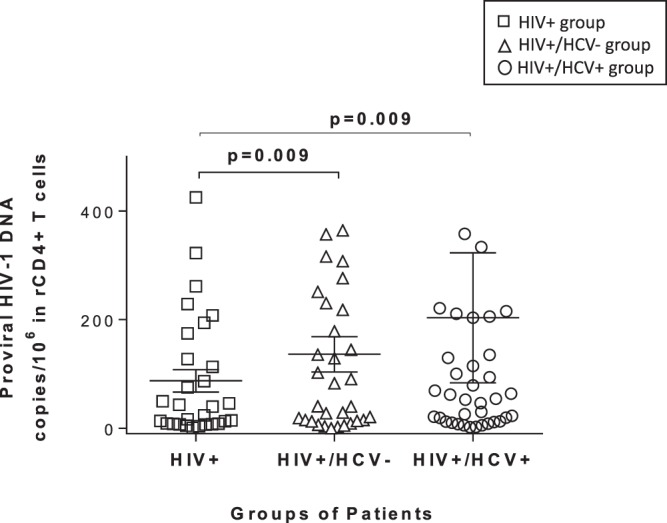
HIV-1 DNA proviral integration. Integrated HIV-DNA in resting CD4+ (CD4 + CD25-CD69-HLA-DR-) T lymphocytes from study patients. Integrated HIV DNA was quantified by q-PCR in cross-sectional samples from different groups of patients: (1) HIV+ group: HIV-monoinfected patients that had never been in contact with HCV (negative PCR and HCV antibodies); (2) HIV+/HCV− group: HIV patients who had been exposed to HCV but experienced spontaneous viral clearance during the first 6 months after HCV infection (negative PCR and positive HCV antibodies); (3) HIV+/HCV+ group: patients with HIV and active HCV-chronic infection naïve to any HCV treatment (positive PCR and HCV antibodies). All patients were under successfully cART. Molecular forms of HIV were amplified by quadruplicated and values are means ± standard error of the mean (SEM). Generalized linear models (GLM) and gamma distribution (log-link) were used to evaluate differences of number of copies of integrated HIV-DNA among study groups. These models were adjusted by main clinical and epidemiological variables (see Materials and Methods). Numbers on the bars are the *p*-values in the multivariant analysis. P values showed belongs to the multivariant analysis.

Mean of proviral HIV-1 DNA copies/10^6^ in rCD4+ T cells was 87.34 ± 22.36 copies in HIV+ patients, 206.21 ± 47.38 copies in HIV+/HCV+ patients and 136.20 ± 33.20 copies HIV-1 in HIV+/HCV− patients (Fig. 1). In HIV+/HCV+ coinfected patients, a 2.3-fold increase in proviral HIV-1 DNA copies was observed as compared to HIV-monoinfected group (*P* = 0.013). This difference remains statistically significant (*P* = 0.009) in the multivariate analysis, adjusting by age, gender, total and nadir CD4 cells, transmission route and time of infection (Fig. 1 and Table 4). In HIV+/HCV− patients, the univariate model showed a tendency to increase in the copies number of proviral HIV-DNA (1.6-fold) although it did not show statistical significance (p = 0.209). However, when multivariate analysis was performed, adjusting by gender (male), total nadir CD4, time of HIV infection and transmission route, a statistically significant increase was observed in proviral HIV-DNA copies in HIV+/HCV− subjects as compared to the HIV-monoinfected group (*P* = 0.009) (Fig. 1 and Table 4).

**Table 4:** Linear regression analysis by generalized linear model (GLM): (A) Univariate and (B) multivariate (adjusted) analysis. Differences of the number of copies of integrated HIV-DNA among study groups were evaluated.

Groups	Primary outcome Mean ± SEM	Univariate analysis ARM (95% CI)	P	Multivariate analysis aAMR (95% CI)	P
HIV+	87.34 ± 22.36	0	—	0	—
HIV+/HCV−	136.20 ± 33.20	1.559 (0.780; 3.118)	0.209	2.514 (1.259; 5.022)	0.009
HIV+/HCV+	206.21 ± 47.38	2.361 (1.203; 4.634)	0.013	2.659 (1.273; 5.554)	0.009

Note: (a) Univariate analysis comparing HIV-1 DNA provirus vs HIV+/HCV− group or HIV+/HCV+ groups respectively. (b) Multivariate analysis comparing HIV-1 DNA provirus vs HIV+/HCV− group or HIV+/HCV+ groups respectively after adjustment by adjusted by age, gender, total CD4+ T cells, nadir CD4+ T cells, transmission route and time of infection. SEM: standard error of the mean; 95%CI: Confidence Interval; ARM: Arithmetic mean ratio; aARM: Adjusted arithmetic mean ratio.

Patients: ▪ HIV+ group: HIV-monoinfected patients that had never been in contact with HCV (negative PCR and HCV antibodies).

▪ HIV+/HCV− group: HIV patients who had been exposed to HCV but experienced spontaneous viral clearance during the first 6 months after HCV infection (negative PCR and positive HCV antibodies).

▪ HIV+/HCV+ group: patients with HIV and active HCV-chronic infection naïve to any HCV treatment (positive PCR and HCV antibodies).

### Associations between HIV-1 DNA provirus in rCD4 T-cells and the epidemiological and clinical characteristics in the different patients groups

Finally, we investigated correlations between the number of copies of HIV-1 DNA provirus in rCD4 T-cells and the epidemiological and clinical data in the different group of patients.

In HIV-monoinfected individuals, a negative correlation was observed between proviral load and the infection time (SF1), (ρ = −0.506, p = 0.06), whereas a positive correlation between proviral load and male sex (ρ = 0.408, p = 0.028) was identified. In HIV+/HCV− group, proviral load was associated to the HIV clinical stage (CDC category) (ρ = 0.354, p = 0.47). In HIV+/HCV+ coinfected patients, a negative correlation was found between proviral load and CD4% (ρ = −0.365, p = 0.031), and also between provirus vs nadir CD4% (ρ = −0.583, p = 0.005) (SF2).

Finally, no correlations were found between proviral HIV-DNA in rCD4 T-cells and age, ratio CD4:CD8, transmission route and the different treatment regimens in any group of patients. No correlation was observed either between proviral HIV-DNA and the fibrosis stage in spontaneous clarifiers and HIV/HCV coinfected individuals (SD2).

## Discussion

HIV-1 and HCV infections are major health concerns all over the world. When co-infected, patients show a poor prognosis than during HIV-1 monoinfection^27^. This is particularly important regarding to HIV-1 infection, as this virus forms a persistent, long-lived reservoir that cannot be eliminated with current cART. It has been described that HIV-1 reservoir can be modulated by co-infection with other viruses such as CMV^26^ but no information is available regarding to the effect of HCV coinfection on the HIV-1 reservoir size, despite the fact that 6.2% of HIV-1 infected patients are currently co-infected with HCV^16^. Therefore, to our knowledge, this is the first study that shows how the HIV-1 reservoir size may be modified during the natural history of HCV infection, including HCV spontaneous cleared infection and HCV chronic-infected individuals.

We observed that chronic HCV infection was associated with an increased HIV-1 proviral integration in cART-treated HIV-1 infected patients. Additionally, HIV-1 infected patients who spontaneously clarified HCV showed an association in HIV-1 proviral integration in multivariant analysis. Due to the limited amounts of sample available, the frequency of cells harboring replication-competent virus could not be evaluated. However, integrated HIV-1 DNA in rCD4+ T cells reflects the size of the replication-competent virus in patients receiving ART^28,29^ and is considered an adequate surrogate marker to evaluate the size of the latent reservoir in clinical studies^30,31^.

Previous studies have shown that HIV-1 cell-associated RNA transcription and HIV-1 DNA integration are reduced in HIV/HCV co-infected patients undergoing therapy with IFN-α and ribavirin^32–34^. The mechanism by which this occurs includes increased levels of the restriction factor TRIM22 induced by IFN-α^32^ and improved cytotoxic function of natural killer cells^34^. Therefore, we analyzed the effect of HCV co-infection on HIV-1 proviral DNA in patients who had not received previous HCV therapy and had started cART in the same Fiebig stage (Phase V). This patient selection represented the interaction between HIV-1 and HCV during the natural evolution of HVC infection, in patients with spontaneous HCV clearance or with HCV chronic infection.

HIV-1 reservoir is very stable and remains constant despite the effective cART^3,10^. Maintenance and replenishment of HIV-1 latently infected cells occurs through several mechanisms such as cellular activation and clonal and homeostatic expansion that may be relevant in the eventuality of coinfection^4,35^. In fact, it has been described that immune activation induced after co-infection with pathogens such as *Mycobacterium tuberculosis* enhances HIV-1 progression by increasing the number of activated CD4+ T cells^36,37^. This is due to HIV-1 may infect both resting and activated CD4+ T cells but only replicates in activated cells^4^. Cellular activation may also be responsible for the persistent ongoing viral replication that contributes to maintain the reservoir due to suboptimal drug concentrations in lymphoid organs^38^. Consequently, T cell activation due to persistent HCV replication observed in HCV chronic co-infected patients may likely account for the higher HIV-1 integration observed in these patients. The higher number of intravenous drug users (IDU) enrolled in the groups exposed to HCV may also explain part of these differences. There is an immunostimulatory effect on IDU in HCV chronic infected patients^39^ that may lead to systemic immune activation, making impact on HIV-1 integration. Additionally, clearance of HIV-1 replicating cells after viral reactivation relies on host cytotoxic immune responses that become dysfunctional and exhausted after long exposure to persistent antigenic stimulation, which is more prevalent in HCV chronic patients^40^.

HCV has also been described to infect lymphoid cells with higher frequency in HIV-1 infected patients^41^. The biological significance of lymphotropic HCV has not been fully elucidated yet and is generally assumed that co-infection of HIV-1 latently infected cells, although possible, would be extremely rare due to the low size of HIV-1 reservoir^8^. However, this event may influence on the persistence of HIV-1 reservoir due to HCV may activate the transcription factor NFAT (nuclear factor of activated T-cells) and induce the expression of IL-2^42,43^ and IL-7^44^, being both cytokines involved in homeostatic proliferation that contributes to reservoir replenishment^45^. HIV-1 infected cells also proliferate due to clonal expansion^46,47^ but the influence of HCV on this mechanism has not been described yet. Further studies would be required to solve the relative contribution of homeostatic and clonal expansion in HIV-1 reservoir persistence during HIV-1/HCV co-infection.

We demonstrated that chronic HCV infection may influence on HIV-1 proviral integration. In accordance to our findings, co-infection with other viruses such as CMV also increased HIV-1 DNA levels and presumably reservoir seeding in cART-naïve HIV-1 patients^26^. Co-infection of HIV-1 and CMV shares other similarity with HIV-1 and HCV such as that CD4:CD8 ratio was significantly reduced in HIV-1/CMV coinfected subjects in comparison to HIV-infected CMV-seronegative individuals^20^. Accordingly, we observed that CD4:CD8 ratio also decreased in HIV-1/HCV coinfected patients. Altogether, these data supported that co-infection with other pathogens during HIV-1 disease may increase the reservoir size and affect the disease progression. Despite subjacent molecular mechanisms remain to be elucidated, this should be considered during the management of sexual transmitted diseases frequently concurring in HIV-1 infected patients. Moreover, recent studies suggested that viral latency is not limited to resting and quiescent CD4+ T cells but that activated and proliferating cells may also contribute to the reservoir^48,49^. Therefore, the number and type of CD4+ T cells able to support viral latency may be larger than initially assumed. Further studies in these activated cell populations of HIV-1/HCV co-infected patients would seem necessary to ensure that the reservoir is not underestimated, since its depletion is the ultimate goal in HIV cure.

In conclusion, we demonstrated that the reservoir of proviral HIV-1 is enhanced in patients also exposed to HCV in comparison to HIV-monoinfected individuals. This would mean that the elimination of HIV-1 reservoir in HIV/HCV-coinfected individuals could be even more complex.

## Methods

### Patients

Cross-sectional study of 97 European HIV-1 infected adults receiving suppressive cART for at least one year. Patients were enrolled in five tertiary hospitals from the Madrid region (Spain) (SD1). Samples were collected between November 2016 and June 2017. All patients had been undetectable for HIV during the previous year and had CD4+ T-cells ≥500 cells/mm^3^ since at least one year before sample collection. Exclusion criteria for patient selection were pregnancy, hepatic decompensation, liver damage due to alcohol intake, presence of viral hepatitis B antigens or antibodies against viral hepatitis B, opportunistic infections, substance abuse, other diseases: diabetes, nephropathies, autoimmune diseases, hemochromatosis, cryoglobulinemia, primary biliary cirrhosis, Wilson’s disease, deficiency of alpha1 antitrypsin and neoplasia. Evaluation of liver fibrosis was performed by transient elastography (TE) (FibroScan-502® (Echosens, Paris) using the following cut-off values for fibrosis of kPa to identify the different stages of fibrosis: F0-F1 (<6kPA), F2 (6–9kPa), F3 (>9–12kPa) and F4 (>12kPa)^50^. IFNL3 rs12979860 single nucleotide polymorphism analysis was performed by qPCR using a custom taqman polymorphism assay (Life Technologies, California, USA) for rs12979860. Plasma HCV RNA viral load was measured by COBAS® TaqMan® HCV Test v2.0 (Roche Diagnostic Systems Inc., Branchburg, New Jersey, USA) and plasma HIV-1 RNA viral load was measured by Amplicor Monitor assay (Roche Diagnostic Systems Inc., Branchburg, New Jersey, USA) and real-time NASBA (Easy Mag y Nuclisens Easy Q; BioMerieux, Marcy l′Etoile, France) with a detection limit of 20 copies/ml (undetectable viral load).

Age, weight, height, BMI, CDC classification system for HIV infection, cART regimens, CD4 and CD8 T cell counts, Nadir CD4 + , HCV genotype, fibrosis status and CMV infection, were obtained from medical records.

Patients were categorized in 3 groups: (1) HIV+ group (n = 29): HIV-monoinfected patients that had never been in contact with HCV (negative HCV PCR and HCV antibodies); (2) HIV+/HCV− group (n = 32): HIV patients who had been exposed to HCV but experienced spontaneous viral clearance during the first 6 months after HCV infection (negative HCV PCR and positive HCV antibodies); (3) HIV+/HCV+ group (n = 36): patients with HIV and active HCV-chronic infection naïve to any HCV treatment (positive HCV PCR and HCV antibodies). The STROME-ID checklist was used to strength the design and conduct the study^51^.

### Isolation of rCD4+ T cells

Peripheral blood mononuclear cells were extracted from 50 mL of blood and rCD4+ T-cells were isolated by negative selection with immunomagnetic beads using EasyStep Human Resting CD4+ T Cell Isolation kit (StemCell Technologies). The purity of CD4 + CD25−CD69−HLA/DR−T cells was assessed by flow cytometry as >99%.

### Quantification of HIV-1 proviral DNA integration

Total DNA from isolated rCD4+ T cells was extracted using DNeasy Blood & Tissue kit (Qiagen) and the integrated HIV-DNA was measured by nested Alu–HIV-LTR PCR^52^. Briefly, Alu-1, Alu-2 and LTR L-M667 primers and Taq DNA polymerase (Roche) were used for the first conventional PCR; and LTR AA55M, Lambda T primers and MH603 primers and Taqman Universal PCR Master Mix (Promega) for the second quantitative PCR (StepOne, Applied Biosystems). A standard curve of integrated HIV-DNA from 8E5 cell line using serial dilutions was prepared as reference and CCR5 was used as housekeeping gene. Values of 2-LTR circle DNA molecules in rCD4+ T cells were subtracted from the integrated HIV-DNA value. Primary outcome was the proviral HIV-1 DNA/10^6^ rCD4 T cells. Results were given in copy of HIV-1 DNA integrated/rCD4 + cell.

### Statistical Analysis

The baseline characteristics of the study population were presented as percentages for categorical variables and as medians and interquartile ranges (IQR) for continuous variables. The normalized status of the different variables included was verified through the Kolmogorov-Smirnov test. χ^2^ test and the 2-sided Fisher exact test (non-parametric) were used to compare categorical variables. Kruskal-Wallis H test and Mann Whitney U test were used to compare continuous variables. Correlation between the variable of interest (proviral HIV-1 DNA) and other quantitative variables was analyzed using the Spearman rank correlation test.

Univariate and multivariate analysis with generalized linear models (GLM), gamma distribution (log-link), were used to evaluate differences of the number of copies of integrated HIV-DNA among study groups. The number of copies are given in mean and standard error of mean (SEM) for each group. This test gives the arithmetic mean ratio (AMR) or the value by which the arithmetic mean of the primary outcome is multiplied. Each multivariate regression test was adjusted by age, gender, total CD4+ T cells, nadir CD4+ T cells, transmission route and time of infection. SPSS (v.17; SPSS Inc., Chicago, IL) was used. Statistical significance was defined as P < 0.05 (2-tailed).

### Ethics

An informed consent was obtained from each subject. Confidentiality and privacy was assured. The protocol study was approved by review broads from each institution involved [*Comité de Ética de la Investigación* of Institute of Health Carlos III (Ref: CEI PI67_2015-v4), *Comité de Ética de la Investigación* of *Hospital La Paz* (Ref: PI-2601), *Secretaría Técnica del Comité de Ética de Investigación* of *Hospital Universitario 12 de Octubre* (Ref: 16/223), *Secretaría del Comité de ética de Investigación Médica of Hospital La Princesa (*Ref: PI15CIII/00031), *Comité Ético de Investigación Clínica of Hospital General Universitario Gregorio Marañón* (Ref: 09/2016), *Comité Ético de Investigación con Medicamentos* of *Hospital Puerta de Hierro* (Ref: 20.16)] and developed in accordance to the Helsinki Declaration, as revised in 2013.

## Supplementary information

Supplementary Information
