# Supplementary material for: HCV-coinfection is related to an increased HIV-1 reservoir size in cART-treated HIV patients: a cross-sectional study
Source: Sci Rep. 2019 Apr 3;9:5606. doi: 10.1038/s41598-019-41788-9 (PMC6447590; doi:10.1038/s41598-019-41788-9)

Type of manuscript: Original

Title: HCV-coinfection is related to an increased HIV-1 reservoir size in cART-treated HIV patients: a cross-sectional study.

Running head: HCV-coinfection increases HIV-1 reservoir size

Authors: Mª Rosa LÓPEZ-HUERTAS[1], Claudia PALLADINO[2], Marta GARRIDO-ARQUERO[3], Beatriz ESTEBAN-CARTELLE[3], Marta SÁNCHEZ-CARRILLO[3], Paula MARTÍNEZ-ROMÁN[3], Luz MARTÍN-CARBONERO[4], Pablo RYAN[5], Lourdes DOMÍNGUEZ-DOMÍNGUEZ[6], Ignacio DE LOS SANTOS[7], Sara DE LA FUENTE MORAL[8], José Miguel BENITO[9,10], Norma RALLÓN[9,10], José ALCAMÍ[11], Salvador RESINO[3], Amanda FERNÁNDEZ-RODRÍGUEZ[3¶], Mayte COIRAS[11], Verónica BRIZ[3], on behalf of the Multidisciplinary Group of viral coinfection HIV/Hepatitis (COVIHEP)*

[1]Department of Infectious Diseases, Instituto Ramón y Cajal de Investigación Sanitaria (IRYCIS), Madrid, Spain.

[2]Research Institute for Medicines (iMed.ULisboa), Faculty of Pharmacy, Universidade de Lisboa, Lisbon, Portugal.

[3]Unit of Viral Infection and Immunity, National Center for Microbiology, Institute of Health Carlos III, Majadahonda, Madrid, Spain.

[4]Instituto de Investigación Sanitaria Hospital de la Paz (IdiPAZ), Madrid, Spain.

[5]Department of Infectious Diseases, Infanta Leonor Hospital, Madrid, Spain.

[6]Unidad VIH. Servicio de Medicina Interna. Instituto de Investigación Biomédica del Hospital Doce de Octubre (imas12), Madrid, Spain.

[7]Servicio de Medicina Interna-Infecciosas. Hospital Universitario de La Princesa, Madrid, Spain.

[8]Servicio de Medicina Interna. Hospital Puerta de Hierro, Madrid, Spain.

[9]Instituto de Investigación Sanitaria Fundación Jiménez Díaz, Universidad Autónoma de Madrid (IIS-FJD, UAM), Madrid, Spain.

[10]Hospital Universitario Rey Juan Carlos, Móstoles, Spain.

[11]AIDS Immunopathology. Centro Nacional de Microbiología, Instituto de Salud Carlos III, Majadahonda, Madrid, Spain.

¶ **Corresponding author:**

Amanda Fernárnez-Rodríguez

Institute of Health Carlos III

National Center of Microbiology

Carretera Majadahonda-Pozuelo km 2.2

28220-Majadahonda (Madrid)

Spain

Email: amandafr@isciii.es

Telephone number: 0034918223892

Fax number: 0034915097966

Alternative corresponding author:

Verónica Briz

Institute of Health Carlos III

National Center of Microbiology

Carretera Majadahonda-Pozuelo km 2.2

28220-Majadahonda (Madrid)

Spain

Email: veronica.briz@isciii.es

Telephone number: 0034918223892

Fax number: 0034915097966

Supplemental Data 1. Hospitals participating in this study.

Hospital Universitario La Paz, Madrid (41 patients); Hospital Universitario Doce de Octubre, Madrid (26 patients); Hospital Universitario Infanta Leonor, Madrid (17 patients); Hospital Universitario La Princesa, Madrid (8 patients); Hospital Universitario Puerta de Hierro, Madrid (5 patients).

Supplemental Data 2. Correlations between the HIV-1 proviral integration in rCD4 T cells and different epidemiological and clinical data.

Epidemiological and Clinical data			Copies of HIV-1 DNA provirus/10^6 rCD4 T cells			
			All patients	HIV+ group	HIV+/HCV- group	HIV+/HCV+ group
Spearman's rho	Age (years)	Correlation Coefficient	0.034	-0.087	0.080	0.099
		Sig. (2-tailed)	0.742	0.654	0.663	0.568
		N	97	29	32	36
Spearman's rho	Height (cm)	Correlation Coefficient	-0.059	0.209	0.071	-0.423
		Sig. (2-tailed)	0.610	0.350	0.732	***0.020***
		N	78	22	26	30
Spearman's rho	Time of HIV infection (years)	Correlation Coefficient	-0.041	-0.506	0.087	0.092
		Sig. (2-tailed)	0.695	0.006	0.634	0.592
		N	96	28	32	36
Spearman's rho	CD4+ T cell count	Correlation Coefficient	-0.091	-0.194	0.076	-0.113
		Sig. (2-tailed)	0.377	0.313	0.680	0.513
		N	97	29	32	36
Spearman's rho	CD4+ %	Correlation Coefficient	-0.112	-0.125	0.065	-0.174
		Sig. (2-tailed)	0.275	0.517	0.722	0.318
		N	96	29	32	35
Spearman's rho	Nadir CD4	Correlation Coefficient	-0.150	-0.184	-0.130	-0.133
		Sig. (2-tailed)	0.181	0.414	0.518	0.467
		N	81	22	27	32
Spearman's rho	Nadir CD4 %	Correlation Coefficient	-0.163	0.113	-0.051	-0.583
		Sig. (2-tailed)	0.233	0.714	0.825	***0.005***
		N	55	13	21	21
Spearman's rho	CD8+ T cell count	Correlation Coefficient	0.210	0.274	0.257	0.113
		Sig. (2-tailed)	0.120	0.257	0.319	0.363
		N	56	19	17	20
Spearman's rho	CD8 %	Correlation Coefficient	0.011	0.063	0.038	-0.119
			0.934	0.797	0.882	0.618
			56	19	17	20
Spearman's rho	CD4:CD8 Ratio	Correlation Coefficient	-0.174	-0.332	0.045	-0.181
			0.199	0.165	0.865	0.445
			56	19	17	20

Supplemental Figure 1. Correlations between the HIV-1 proviral integration in rCD4 T cells and time of HIV infection.

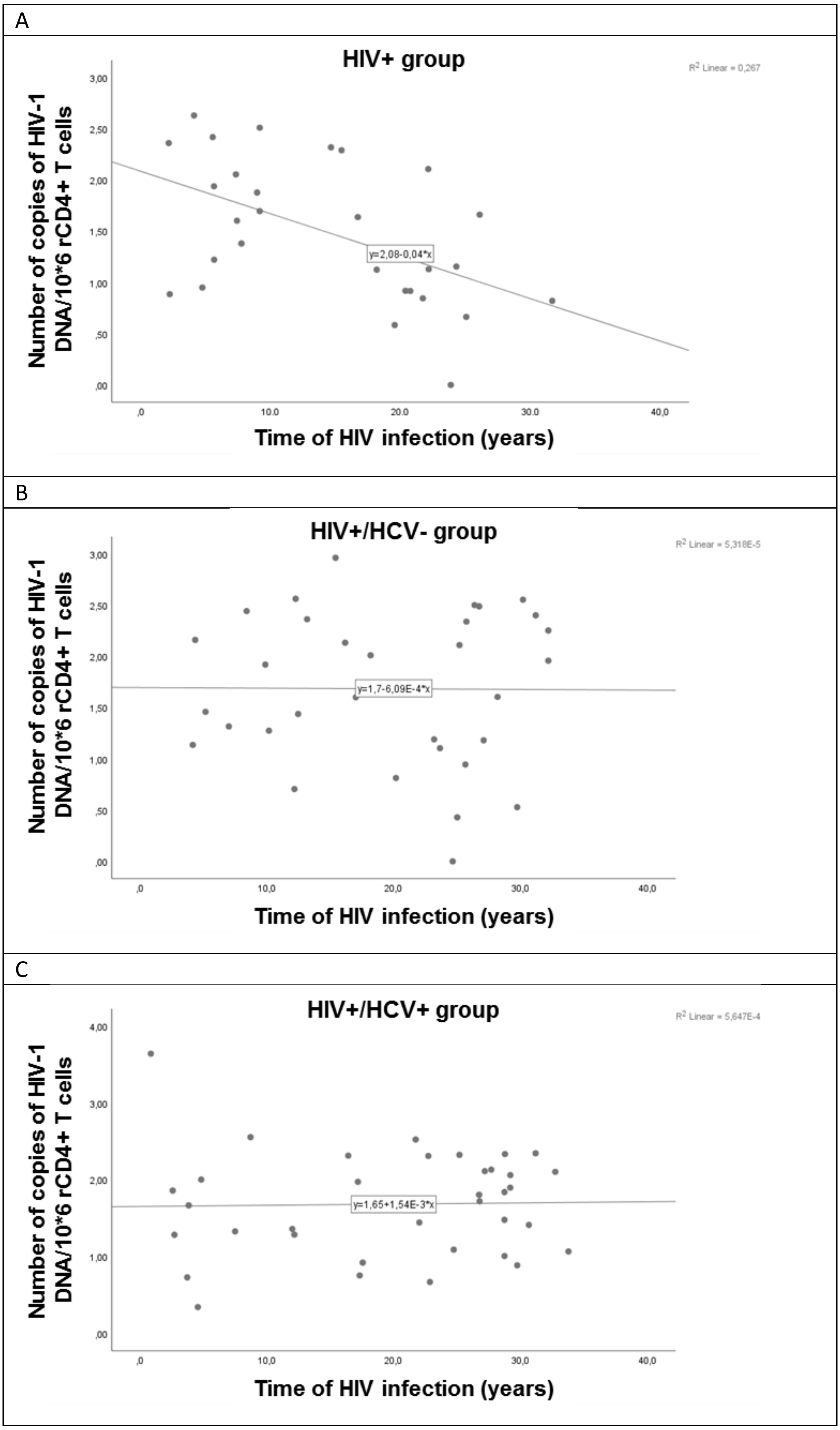

Supplemental Figure 2. Correlations between the HIV-1 proviral integration in rCD4 T cells and nadir of CD4 %.

A

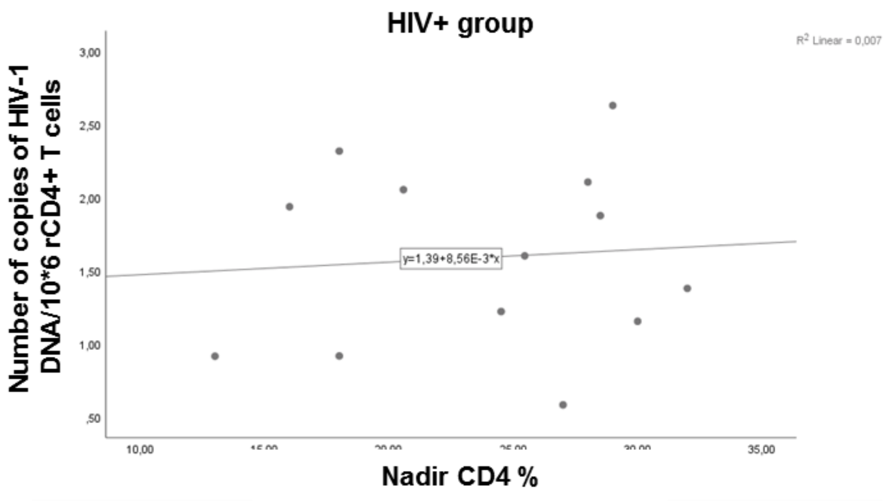

B

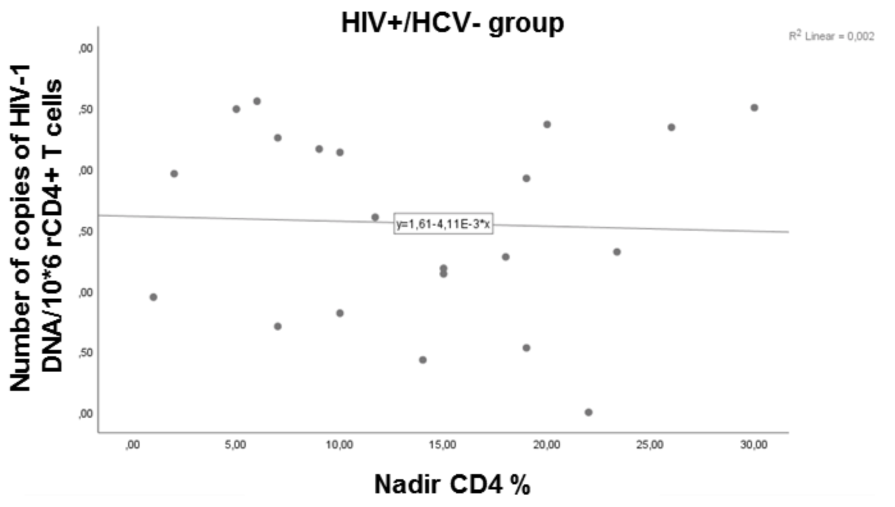

C

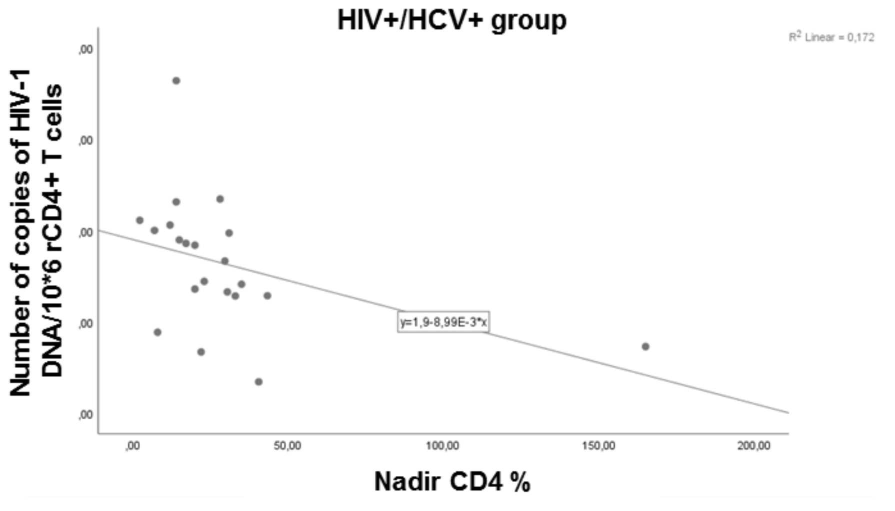

Supplement: Supplementary file 1 — Supplementary Information [file 41598_2019_41788_MOESM1_ESM.pdf]
